# Artificial intelligence advancements for orthopaedic clinical reasoning: longitudinal assessment of newer models (ChatGPT-5, Grok-3, Gemini 2.5 Flash) compared to clinicians

**DOI:** 10.1007/s00402-026-06400-6

**Published:** 2026-07-07

**Authors:** Suzen Agharia, Shayan Soroush, Daniel Ameen, Yushy Zhou

**Affiliations:** 1 Faculty of Medicine, Nursing and Health Sciences, Monash University, Clayton, Australia; 2 Department of Orthopaedic Surgery, St. Vincent’s Hospital, Melbourne, Australia

**Keywords:** Generative AI, Large language models, Orthopaedic decision-making, Clinician consensus, Benchmarking

## Abstract

**Introduction:**

This descriptive study aimed to longitudinally evaluate the performance of contemporary large language models - ChatGPT-5, Gemini 2.5 Flash, and Grok-3 - on orthopaedic clinical multiple-choice tasks, benchmarked against pooled clinician consensus. A secondary aim was to assess whether recent advances in generative AI translated into improved alignment with clinician consensus compared with previous AI models.

**Materials and methods:**

A total of 97 multiple-choice clinical cases spanning eight orthopaedic subspecialties were sourced from OrthoBullets and previously benchmarked against aggregated responses from thousands of practising clinicians. Using identical methodology to our 2023 study of ChatGPT-3.5, ChatGPT-4, and Bard, each model was prompted with standardised case stems and response options. The primary outcome was the proportion of AI responses matching the most popular clinician response; secondary analyses assessed agreement within 10% and 20% of clinician consensus, performance on ‘controversial’ (< 25% margin) questions, and inter-model concordance using Cohen’s kappa coefficients.

**Results:**

Gemini 2.5 Flash achieved the highest alignment with clinician consensus (69.1%), followed by Grok-3 (66.0%) and ChatGPT-5 (58.8%). None of the LLMs refused to respond to any prompts, representing a reduction from 7.2% from our 2023 study. Subspecialty analysis demonstrated that Gemini 2.5 Flash performed best in Hand and Paediatric domains, while Grok-3 excelled in Reconstruction, Trauma, and ‘controversial’ cases. Inter-model agreement was highest between Grok-3 and Gemini 2.5 Flash (κ = 0.678), indicating improved consistency compared with prior-generation systems.

**Conclusions:**

Contemporary LLMs can be promising adjuncts for orthopaedic education by simulating peer reasoning and offering structured explanations in non-critical settings. Despite incremental gains in reasoning capability compared to previous AI models, contemporary LLMs remain unsuitable for independent clinical use. Future research should develop hybrid clinician–AI workflows and longitudinal benchmarks to distinguish true reasoning improvements from memorisation.

**Supplementary Information:**

The online version contains supplementary material available at 10.1007/s00402-026-06400-6.

## Introduction

 Recent advancements in generative artificial intelligence (AI) have accelerated the incorporation of large language models (LLMs) into clinical medicine, education, and decision support [1–4]. A rapidly expanding body of work has explored their applications in diverse domains, including urology, cardiothoracic anaesthesia, solid organ transplantation, paediatrics, trauma, emergency medicine, and surgical admissions, yet their application in orthopaedic clinical decision making remain relatively unclear [4–8]. Collectively, these studies suggest that LLMs can assist clinicians in generating diagnostic and management recommendations, sometimes achieving performance close to expert or guideline-based standards. However, findings remain inconsistent, with several investigations reporting factual inaccuracies, gaps in contextual reasoning, and model variability across specialties [3, 5, 7–9]. Such heterogeneity underscores the need for ongoing research to determine whether technical advances in LLMs reliably translate into meaningful improvements in clinical decision-making, particularly in orthopaedic surgery.

One of the persistent challenges in evaluating AI in healthcare is the lack of standardised benchmarks that reflect real-world clinical reasoning [10]. Many studies have relied on examination-style questions, guideline concordance, or synthetic case vignettes [9, 11–13]. While useful, these approaches often fail to capture the complexity of clinical practice, where multiple reasonable courses of action may coexist and judgement depends on consensus among experienced peers. Consequently, such studies may overestimate model accuracy or fail to identify gaps in contextual understanding. Our initial 2023 study addressed this literature gap by evaluating the ability of ChatGPT-3.5, ChatGPT-4, and Bard to answer orthopaedic clinical cases from the OrthoBullets platform, directly comparing their responses against pooled decisions from thousands of practicing clinicians [14]. This provided a clinically grounded benchmark for evaluating AI reasoning against human consensus rather than binary ‘correct or incorrect’ answers.

The present work builds directly on that foundation. We re-tested the same 97 cases using three next-generation LLMs - ChatGPT-5, Gemini 2.5 Flash, and Grok-3- while keeping the same dataset, methodology, and human comparator data. This study design offers a unique longitudinal opportunity to track how generative AI responses have evolved over time, and whether newer architectures show improved alignment with pooled clinician consensus under controlled, reproducible conditions. Understanding whether newer models display more consistent concordance with peer decision-making has implications for clinician trust and the responsible use of generative AI in healthcare. Our primary aim was to evaluate the alignment of these newer LLMs with orthopaedic expert consensus using a descriptive statistics approach. We also examine how LLM responses have changed since our 2023 evaluation of earlier-generation models.

## Methods

This study was conducted using an identical methodology to our previous publication evaluating ChatGPT-3.5, ChatGPT-4, and Bard in orthopaedic clinical decision-making [14]. A summary of the approach is provided below, with modifications noted for the updated AI tools. Ethical approval was not required, as only publicly available, non-identifiable clinical cases from OrthoBullets were used [15]. Written permission was obtained from OrthoBullets prior to study commencement.

### AI tools

The current study evaluated three updated LLMs: ChatGPT-5 (OpenAI, September 5, 2025 version), Grok-3 (xAI, September 5, 2025 version), and Gemini 2.5 Flash (Google DeepMind, September 5, 2025 version). These were tested in place of the models assessed in our initial study.

### Clinical cases and questions

**Fig. 1:**
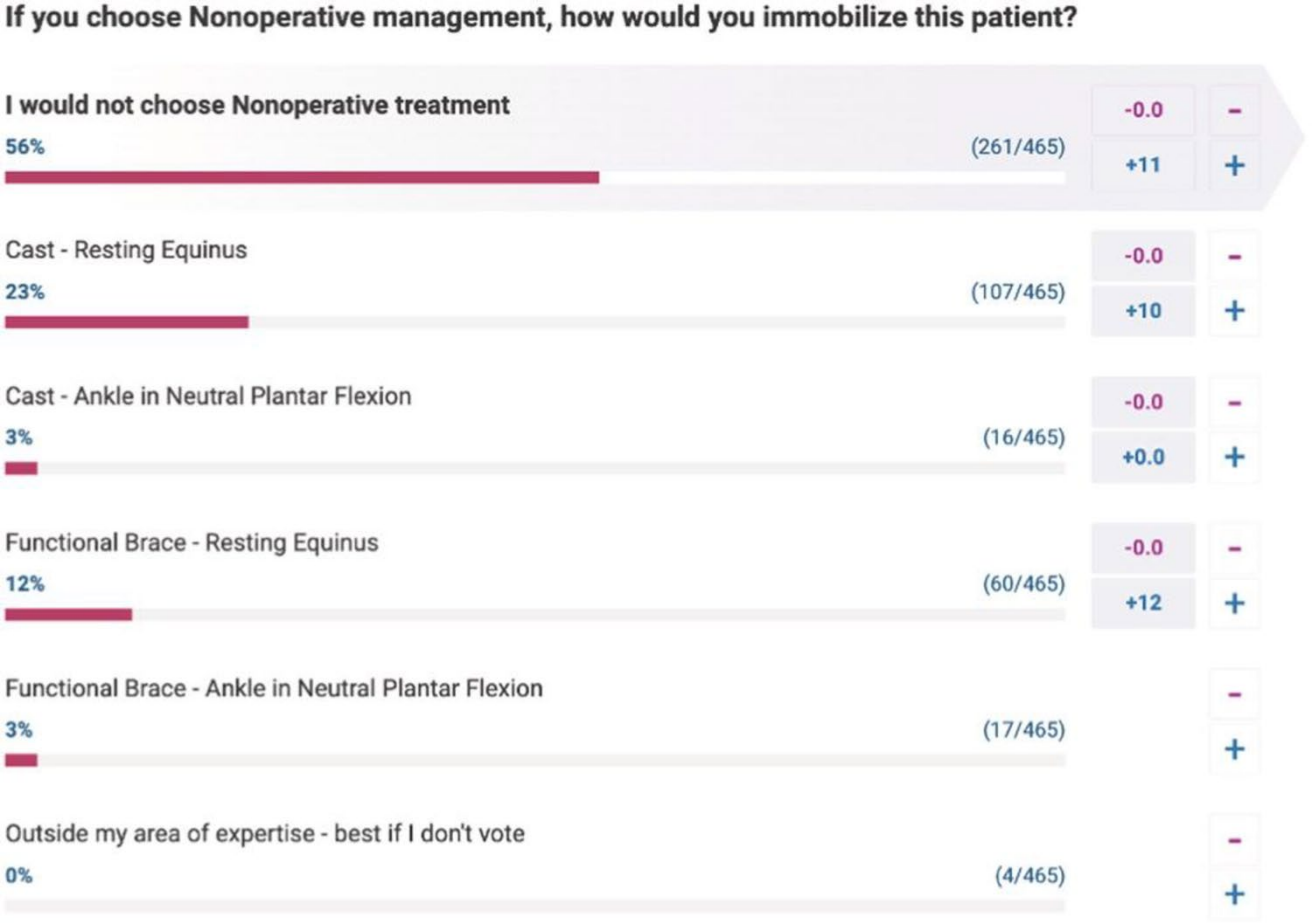
Example screenshot of the questions and response options for clinical cases published on OrthoBullets

As in the prior study, clinical cases were sourced from the OrthoBullets platform on July 21, 2023 [16]. Cases spanned the following orthopaedic subspecialties: foot and ankle, hand, knee and sports, paediatrics, reconstruction, shoulder and elbow, spine, and trauma. For each case, multiple-choice questions addressing diagnostic, management, and perioperative considerations were included (Fig. 1). Unique question stems per major orthopaedic subspecialty were used, ensuring a balanced and comprehensive representation across all domains of orthopaedics. The human comparator dataset consisted of the identical clinical cases and voting distributions obtained in our 2023 study. These were intentionally reused, rather than re-extracted from the OrthoBullets platform, to ensure consistency and permit a true longitudinal evaluation of AI model performance over time.

### Outcome measures

The primary outcome was the proportion of AI responses matching the most popular clinician response, hereafter referred to as alignment with clinician consensus. We note explicitly that this represents agreement with the modal crowdsourced clinician response, not a clinical gold-standard or guideline-derived ground truth. The most popular response in any given case may still be suboptimal, regionally biased, or training-level dependent, and our outcomes should be interpreted as concordance with peer decision-making rather than absolute clinical accuracy. Secondary analyses included:

(i) AI responses within 10% and 20% of the most popular response. For each OrthoBullets question, clinicians selected a multiple-choice option; the percentage choosing each was recorded. An AI response was counted as “within 10%” if its vote share was no more than 10% points below the top answer (e.g., most popular = 60% → AI ≥ 50%). ‘Within 20%’ required no more than 20 points below (e.g., most popular = 60% → AI ≥ 40%). Exact matches to the top response were automatically included in both thresholds.
(ii) Performance on ‘controversial’ questions. Defined as < 25% margin between the top two human responses. Non-controversial questions typically reflect guideline-based recall and are straightforward, while controversial questions require nuanced clinical reasoning and are more ‘human-like’, hence providing a more relevant test of AI capability for our study.
(iii) Agreement between AI models (Cohen’s kappa (κ) coefficient).

### Prompting and data collection

A standardised prompt template (Supplementary 1–3) was applied uniformly across all AI systems. Clinical case descriptions and answer options were transcribed verbatim into each model. When imaging was included, descriptive text was provided (Supplementary 4) as the intention of our study was to assess LLM alignment with clinician consensus on clinical decision-making tasks rather than image interpretation. If a model initially provided multiple answers, a one-time follow-up prompt was used to ensure single-response selection: *“For the purposes of an educational exercise*,* please select one response only.”*

Refusal rate was defined as the proportion of questions for which the AI initially declined to provide a response.

### Statistical analysis

The primary analysis was descriptive, evaluating differences between ChatGPT-5, Gemini 2.5 Flash, and Grok-3 within specific orthopaedic subspecialties. A supplementary analysis was performed using a paired-measure statistical framework. Because all three models were benchmarked against an identical set of 97 clinical cases, observations were treated as matched rather than independent. Cochran’s Q test was used to determine whether differences existed between the three models for each subspecialty domain (Supplementary 5). To account for the increased risk of Type I errors associated with multiple subgroup testing across eight subspecialties, a Bonferroni correction was applied to all raw p-values. This conservative approach ensures that reported findings are robust and minimises the likelihood of identifying significant results due to statistical noise or small sub-sample sizes (*n* = 10–14 per subspecialty). Due to the small sub-sample sizes and risk for type I error, the analysis for Tables 2, 3, 4 were conducted as supplementary rather than included in the main body of the results. Subspecialty findings should therefore be interpreted as exploratory and descriptive. Analysis of Cohen’s κ was also used to measure the agreement of responses between the AI tools. All statistical analyses were conducted using R (version 4.3.1, R Foundation, Indianapolis, USA).

## Results

### All responses

This analysis included 97 questions across eight orthopaedic subspecialties. No model refused to answer any questions (refusal rate: 0.0% for ChatGPT-5, Gemini 2.5 Flash, and Grok-3).

Gemini 2.5 Flash recorded the highest rate of alignment with the most popular clinician response (69.1%), followed by Grok-3 (66.0%) and ChatGPT-5 (58.8%; *P* = 0.305). When assessing proximity to the modal response, Grok-3 achieved the highest proportion within 10% points (76.3%; *P* = 0.405) and within 20% points (86.6%; *P* = 0.617) of the most popular answer. The mean proportion of OrthoBullets members selecting the same response as the AI was the highest for Gemini 2.5 Flash (46.3 ± 24.8) with a median of 47.0 [0, 99.0]. Full results are shown in Table 1.

**Table 1:** Overall performance of AI models in matching clinician consensus

	ChatGPT 5 (*N* = 97)	Grok-3 (*N* = 97)	Gemini 2.5 Flash (*N* = 97)	*P*-value
Did the AI chose the most popular response?				
No	40 (41.2%)	33 (34.0%)	30 (30.9%)	0.305
Yes	57 (58.8%)	64 (66.0%)	67 (69.1%)	
Was the AI response within 10% points of the most popular response?				
No	31 (32.0%)	25 (25.8%)	23 (23.7%)	0.405
Yes	66 (68.0%)	72 (74.2%)	74 (76.3%)	
Was the AI response within 20% points of the most popular response?				
No	18 (18.6%)	16 (16.5%)	13 (13.4%)	0.617
Yes	79 (81.4%)	81 (83.5%)	84 (86.6%)	
What proportion of OrthoBullets members chose the same response as the AI?				
Mean (SD)	43.9 (25.4)	44.5 (24.6)	46.3 (24.8)	1
Median [Min, Max]	39.0 [3.00, 99.0]	44.0 [1.00, 99.0]	47.0 [0, 99.0]	1

**Fig. 2:**
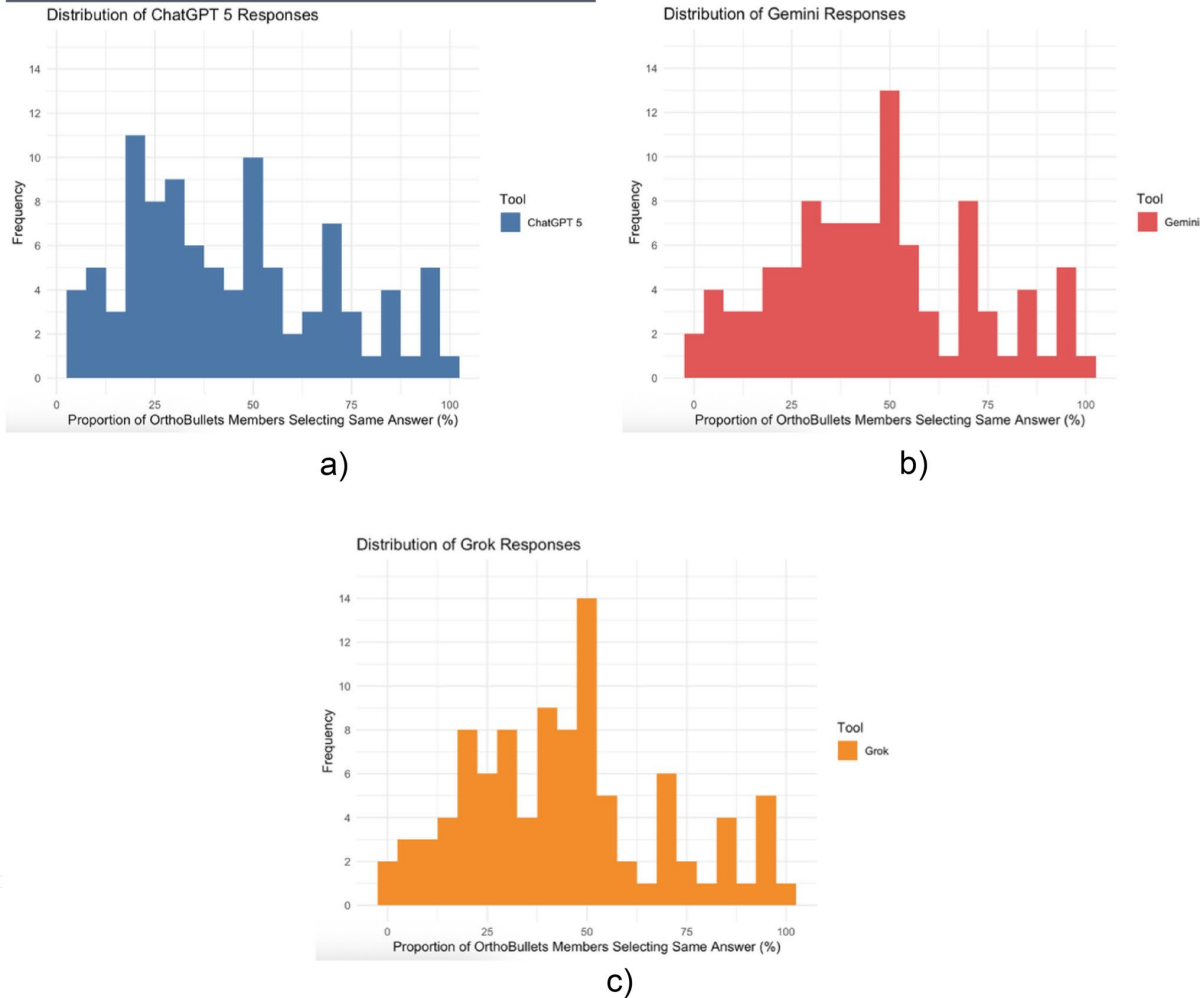
**a**–**c** Distribution of the proportion of OrthoBullets members who voted for the same response as the AI tool response – **a** ChatGPT-5, **b** Gemini 2.5 Flash, and **c** Grok-3

The distribution of clinician agreement with each AI’s selected answer varied widely, from 0% to 99% concordance. Histograms of these proportions are presented for ChatGPT-5 (Fig. 2a), Gemini 2.5 Flash (Fig. 2b), and Grok-3 (Fig. 2c).

### Responses by orthopaedic subspecialty

**Fig. 3:**
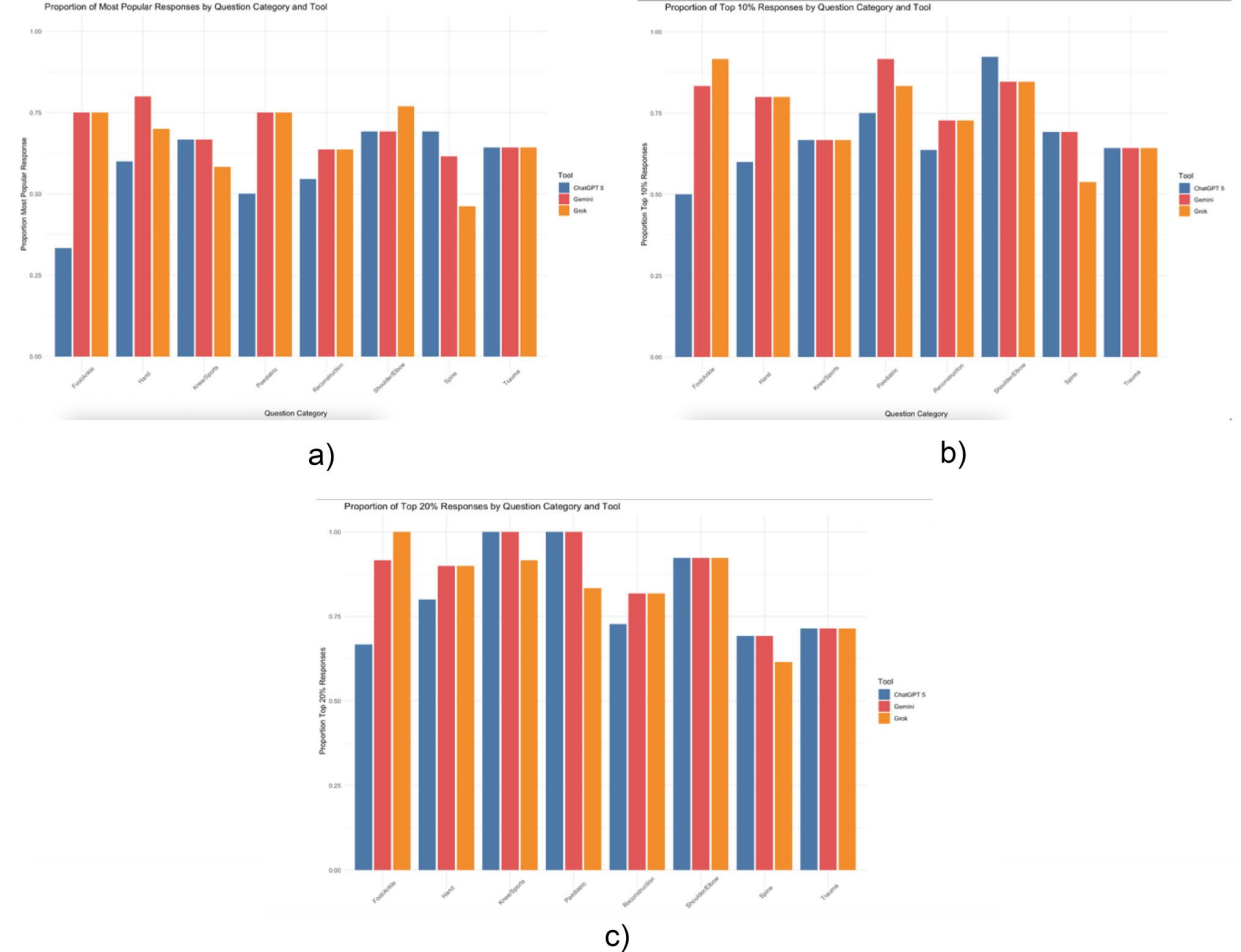
**a**–**c** Clustered bar plot depicting proportions of AI tool responses that aligned with **a** the most popular responses, **b** within 10% of the most popular responses, and **c** within 20% of the most popular responses

Subspecialty performance is detailed in Tables 2, 3, 4 and illustrated in Fig. 3a-c. Gemini 2.5 Flash and Grok-3 tied for the highest rate of most popular responses in Foot/Ankle (both 75.0%). Gemini 2.5 Flash led in Hand (80.0%). Grok-3 outperformed in Shoulder/Elbow (76.9%). In Spine, ChatGPT-5 achieved the highest alignment (69.2%).

**Table 2:** Proportion of AI responses matching the most popular clinician response by subspecialty

Subspecialty	n	ChatGPT-5	Gemini 2.5 Flash	Grok-3
Foot/Ankle	12	33.3%	75.0%	75.0%
Hand	10	60.0%	80.0%	70.0%
Knee/Sports	12	66.7%	66.7%	58.3%
Paediatric	12	50.0%	75.0%	75.0%
Reconstruction	11	54.5%	63.6%	63.6%
Shoulder/Elbow	13	69.2%	69.2%	76.9%
Spine	13	69.2%	61.5%	46.2%
Trauma	14	64.3%	64.3%	64.3%

For responses within 10% of the most popular answer, Grok-3 had the highest concordance in Foot/Ankle (91.7%) and Reconstruction (72.7%). Gemini 2.5 Flash led in Hand, Knee/Sports, and Paediatric (Table 3, Fig. 3b).

**Table 3:** Proportion of responses by tool and category within 10% of top response

Subspecialty	ChatGPT-5	Gemini 2.5 Flash	Grok-3
Foot/Ankle	50.0%	83.3%	91.7%
Hand	60.0%	80.0%	80.0%
Knee/Sports	66.7%	66.7%	66.7%
Paediatric	75.0%	91.7%	83.3%
Reconstruction	63.6%	72.7%	72.7%
Shoulder/Elbow	92.3%	84.6%	84.6%
Spine	69.2%	69.2%	53.8%
Trauma	64.3%	64.3%	64.3%

Within 20% of the most popular response, Grok-3 achieved perfect alignment in Foot/Ankle (100%) and strong performance in Reconstruction. All models were equivalent in Shoulder/Elbow and Trauma. Gemini 2.5 Flash and ChatGPT-5 tied in Knee/Sports and Paediatric (Table 4, Fig. 3c).

**Table 4:** Proportion of responses by tool and category within 20% of top response

Subspecialty	ChatGPT-5	Gemini 2.5 Flash	Grok-3
Foot/Ankle	66.7%	91.7%	100%
Hand	80.0%	90.0%	90.0%
Knee/Sports	100%	100%	91.7%
Paediatric	100%	100%	83.3%
Reconstruction	72.7%	81.8%	81.8%
Shoulder/Elbow	92.3%	92.3%	92.3%
Spine	69.2%	69.2%	61.5%
Trauma	71.4%	71.4%	71.4%

### Sensitivity analysis – controversial and non-controversial questions

In total, there were 46 questions deemed controversial and 51 questions deemed non-controversial. Gemini 2.5 Flash had a higher alignment in non-controversial questions across most categories. In controversial questions, Grok-3 achieved the highest alignment with the most popular response in Reconstruction (75.0%), Trauma (75.0%), and Paediatric (71.4%). Due to smaller subsample sizes, formal statistical testing was not performed (Table 5).

**Table 5:** Proportion of most popular responses by tool and category

Category	Controversial Questions Only (N = 46)			Non-Controversial Questions Only (N = 51)		
	ChatGPT-5	Gemini 2.5 Flash	Grok-3	ChatGPT-5	Gemini 2.5 Flash	Grok-3
Foot/Ankle	20.0%	40.0%	40.0%	42.9%	100%	100%
Hand	40.0%	60.0%	40.0%	80.0%	100%	100%
Knee/Sports	55.6%	55.6%	44.4%	100%	100%	100%
Paediatric	14.3%	57.1%	71.4%	100%	100%	80.0%
Reconstruction	25.0%	50.0%	75.0%	71.4%	71.4%	57.1%
Shoulder/Elbow	50.0%	50.0%	62.5%	100%	100%	100%
Spine	75.0%	75.0%	25.0%	66.7%	55.6%	55.6%
Trauma	50.0%	75.0%	75.0%	70.0%	60.0%	60.0%

### Inter-tool agreement

Inter-model agreement, measured using Cohen’s kappa, ranged from fair (κ 0.21–0.40) to substantial (κ 0.61–0.80). The strongest concordance was between Grok-3 and Gemini 2.5 Flash (κ = 0.678 for most popular response), indicating shared decision patterns. Agreements involving ChatGPT-5 were consistently lower (Table 6).

**Table 6:** Cohen’s kappa coefficients for inter-tool agreement of responses

Comparison	Most popular	Within 10%	Within 20%
Grok-3 vs. Gemini 2.5 Flash	0.678	0.572	0.441
ChatGPT-5 vs. Grok-3	0.512	0.458	0.420
ChatGPT-5 vs. Gemini 2.5 Flash	0.479	0.400	0.335

## Discussion

This study provided a longitudinal evaluation of LLM performance on orthopaedic multiple-choice cases. Using an identical dataset and methodology to our 2023 analysis of ChatGPT-3.5, ChatGPT-4, and Bard, we directly compared newer systems – ChatGPT-5, Gemini 2.5 Flash, and Grok-3 – to determine whether recent advances translated into improved alignment with pooled clinician consensus [14].

Gemini 2.5 Flash achieved the highest alignment with pooled clinician consensus (69.1%), particularly in non-controversial, high-agreement scenarios, where it matched or exceeded the performance of ChatGPT-4 from our prior study [14]. Grok-3 had the highest alignment in controversial cases, especially in Reconstruction, Trauma, and Paediatric orthopaedics, where uncertainty and multiple defensible answers were common. None of the models refused to answer any question, contrasting with the 7.2% refusal rate observed previously. Inter-tool agreement also strengthened (κ = 0.678 for Grok-3 vs. Gemini 2.5 Flash, compared with κ = 0.404 for ChatGPT-4 vs. Bard), suggesting more consistent agreement between newer models on the same items [17–22]. Although the aggregate improvement in top-response alignment did not reach statistical significance, the direction of change was consistently positive across all tools. This growing alignment may reflect convergence in training corpora and instruction-tuning approaches across vendors, although our study was not designed to directly investigate the underlying mechanisms.

We hypothesise that Gemini’s strong performance in non-controversial questions may be related to its known architectural optimisations for logic and structured problem-solving, which heavily favour factual retrieval [8, 18, 19, 23–25]. Conversely, Grok-3’s comparative strength in controversial cases might reflect training paradigms centred on probabilistic reasoning and conversational adaptability [18, 19, 23, 26]. These suggestions remain speculative; our study was not designed to evaluate model architecture, training corpus, or underlying mechanism, and these proposed explanations should be regarded as hypotheses for future targeted investigation rather than conclusions established by our data. Nevertheless, ambiguous cases are arguably the most relevant for assessing reasoning under uncertainty, an area where earlier LLMs consistently underperformed [18, 19, 27, 28].

Our findings mirror broader trends showing that LLMs perform best on algorithmic reasoning tasks and less reliably when implicit spatial or visual interpretation is required [9, 29]. Urda-Cîmpean et al. evaluated the accuracy of diagnostic capabilities of LLMs and noted that all models performed better on knowledge-based items than on reasoning dependent tasks (P = < 0.001) [19]. Similarly, Zhang et al. reported wide variability in orthopaedic LLM performance, with diagnostic accuracy across studies ranging from 55% to 93% and examination-style scores from 45% to 73.6%, highlighting persistent inconsistency in reasoning-heavy tasks [10]. Models can approximate expert reasoning when uncertainty is moderate, as seen in examination-style questions but their performance declines when abstract prioritisation or competing options are involved [6]. These characteristics reinforce the potential value of LLMs in educational contexts, where alignment with typical reasoning is beneficial, but also their current unsuitability for unsupervised clinical decision support [22].

It is important to acknowledge the boundaries of what our methodology can and cannot demonstrate. Performance on multiple-choice clinical vignettes is a widely accepted surrogate for clinical reasoning in medical education and board certification, where synthesising case information, weighing competing variables, and selecting the most appropriate management plan from a list of plausible alternatives forms the basis of standardised assessment. Our use of ‘controversial’ questions, which require navigating genuine ambiguity rather than recall of a single correct fact, was specifically intended to probe this aspect of model behaviour. Nonetheless, agreement with the modal clinician response on archived multiple-choice items is not equivalent to demonstrating reasoning quality, clinical safety, or real-world clinical validity. The present design also cannot fully isolate reasoning capability from the effects of prior exposure to similar content, benchmark familiarity, or improvements in answer formatting. Our findings should therefore be interpreted as evidence of improved concordance with human clinical consensus, rather than as proof of improved reasoning, safety, or clinical decision-making in interactive practice.

A key strength of this study is the replication of the original case set rather than extracting new questions from the OrthoBullets platform. This approach prevented data contamination, as new OrthoBullets material may have been included in LLM training through web scraping or user interactions [17, 30–32]. Although the clinical cases remain on the OrthoBullets platform, the original question stems are no longer publicly available. By using archived, non-public version of these questions, we aimed to minimise the possibility of models recognising the questions and ‘cheating’ by regurgitating memorised answers. This approach enhances the internal validity of our study and allows for temporal comparison across model generations, which is increasingly recommended in AI validation studies [23, 33]. Additionally, evaluating multiple next-generation LLMs under identical conditions allowed meaningful cross-model comparisons, revealing both individual performance differences and shared reasoning trends. Benchmarking model responses against pooled clinician consensus provided a human-clinician standard of comparison, strengthening the clinical relevance of our findings. Finally, categorising questions as controversial or non-controversial enabled exploration of how each model performed under uncertainty, offering insight into where such tools may be most applicable in real-world clinical settings.

Several limitations must be acknowledged. Firstly, while reusing the same cases allowed direct comparison across models, it also limits how much further improvement could be measured on a fixed test set. Furthermore, the LLMs used were not designed for specific clinical decision making. However, they were used given their popularity and accessibility in the real world. The consistent prompt standardisation may also not reflect real-world clinical workflows, where iterative clarification is common [29, 34, 35]. Additionally, the imaging interpretation relied on textural descriptions, not direct analysis, which may introduce potential bias but also mirrors the current LLM constraints. Furthermore, the limitations regarding the small sample size have already been discussed in the “Statistical Analysis” section of this study. Finally, as discussed in Methods, the OrthoBullets crowdsourced consensus is a pragmatic but imperfect benchmark; the modal response is not necessarily guideline-adherent or clinically optimal, and may reflect regional or training-level biases inherent to its contributor population.

In conclusion, this study provides one of the first longitudinal comparisons of newer LLMs in orthopaedic clinical reasoning. Gemini 2.5 Flash and Grok-3 performed comparably and surpassed ChatGPT-5, with Grok-3 showing greater strength in ambiguous, high-uncertainty cases. While encouraging, these gains remain incremental rather than transformative. LLMs are increasingly capable of reflecting clinician decision patterns but remain unsuitable for independent clinical use. Their current value lies in structured decision support and education, where models like Grok-3 and Gemini 2.5 Flash can simulate peer reasoning and guide interactive learning. Future work should focus on hybrid clinician–AI workflows, real-time clinical validation, and longitudinal benchmarking to distinguish genuine reasoning advances from memorisation.

## Supplementary Information

Below is the link to the electronic supplementary material.

Supplementary Material 1

Supplementary Material 2

Supplementary Material 3

Supplementary Material 4

Supplementary Material 5

## Data Availability

No datasets were generated or analysed during the current study.
