# Supplementary material for: Artificial intelligence advancements for orthopaedic clinical reasoning: longitudinal assessment of newer models (ChatGPT-5, Grok-3, Gemini 2.5 Flash) compared to clinicians
Source: Arch Orthop Trauma Surg. 2026 Jul 7;146(1):248. doi: 10.1007/s00402-026-06400-6 (PMC13342123; doi:10.1007/s00402-026-06400-6)
Supplement: Supplementary file 4 — Supplementary Material 4 [file 402_2026_6400_MOESM4_ESM.docx]

Supplementary 4 – Textual descriptors used to communicate findings on images within the clinical cases.

Imaging Descriptors

1. Trauma (27M Femoral Shaft Fracture)

Plain AP and lateral x-ray radiographs show a left comminuted femoral shaft fracture. Plain AP pelvis and AP left knee do not reveal additional injuries.

1. Spine (45F L2 Burst Fracture)

CT and MRI images are provided, showing an L2 burst fracture with retropulsion.

1. Shoulder and Elbow (38F Anterior Shoulder Instability)

Plain (axillary lateral, grashey, and outlet) x-ray radiographs of the right shoulder show subtle signs of a hill-sachs lesion. This is confirmed on MRI scan which shows a hill-sachs lesion (max width 24mm) with shoulder effusion. Furthermore, there is mild to moderate bone loss on the glenoid side. Rotator cuffs are intact.

1. Knee and Sports (31M Patella Tendon Rupture)

Plain AP and lateral x-ray radiographs of the L knee show patella alta with no fractures identified.

1. Paediatric (9F Supracondylar Fracture)

Plain AP and lateral x-ray radiographs show a Gartland 3 left supracondylar fracture.

1. Reconstruction (86F Periprosthetic Hip Fracture)

Plain AP and lateral x-ray radiographs show a likely Vancouver B2 right hip periprosthetic fracture. Previous cemented right total hip arthroplasty is noted.

1. Hand (22M 5^th^ Metacarpal Fracture)

Plain AP, lateral and oblique x-ray radiographs of the left hand show an isolated 5^th^ metacarpal neck fracture, with approximately 45 degrees of dorsal angulation.

1. Foot and Ankle (63F Achilles Tendon Rupture)
   Plain lateral x-ray radiograph of the right foot taken 1 month before the injury shows a calcaneal spur but no acute injury. Plain lateral x-ray radiograph of the right foot taken 1 week before the procedure shows the calcaneal spur has now avulsed, no other acute injury. MRI (single slice, sagittal) of the right foot taken 1 week before the procedure shows the achilles tendon rupture at the insertion of the calcaneus.
