# Supplementary material for: Artificial intelligence advancements for orthopaedic clinical reasoning: longitudinal assessment of newer models (ChatGPT-5, Grok-3, Gemini 2.5 Flash) compared to clinicians
Source: Arch Orthop Trauma Surg. 2026 Jul 7;146(1):248. doi: 10.1007/s00402-026-06400-6 (PMC13342123; doi:10.1007/s00402-026-06400-6)
Supplement: Supplementary file 5 — Supplementary Material 5 [file 402_2026_6400_MOESM5_ESM.docx]

**Supplementary 5 - Paired-measure subspecialty re-analysis (Supplementary Tables 2a–c)**

To rigorously evaluate the differences in performance across ChatGPT-5, Gemini 2.5 Flash, and Grok-3 within specific orthopaedic subspecialties, we employed a paired-measure statistical framework. Because all three models were benchmarked against an identical set of 97 clinical cases, observations were treated as matched rather than independent. Cochran’s Q test was utilised to determine if statistically significant differences existed between the three models for each subspecialty domain. To account for the increased risk of Type I errors associated with multiple subgroup testing across eight subspecialties, a Bonferroni correction was applied to all raw p-values. This conservative approach ensures that reported findings are robust and minimises the likelihood of identifying "significant" results due to statistical noise or small sub-sample sizes (n=10–14 per subspecialty).

**Supplementary Table 2a.** Proportion of AI responses matching the most popular clinician response, by orthopaedic subspecialty, with Cochran’s Q test p-values adjusted using a Bonferroni correction across the eight subspecialties.

| **Subspecialty** | **n** | **ChatGPT-5** | **Gemini 2.5 Flash** | **Grok-3** | **P value (Adjusted)** |
| --- | --- | --- | --- | --- | --- |
| **Foot/Ankle** | 12 | 33.3% | 75.0% | 75.0% | 0.054 |
| **Hand** | 10 | 60.0% | 80.0% | 70.0% | 1.000 |
| **Knee/Sports** | 12 | 66.7% | 66.7% | 58.3% | 1.000 |
| **Paediatric** | 12 | 50.0% | 75.0% | 75.0% | 1.000 |
| **Reconstruction** | 11 | 54.5% | 63.6% | 63.6% | 1.000 |
| **Shoulder/Elbow** | 13 | 69.2% | 69.2% | 76.9% | 1.000 |
| **Spine** | 13 | 69.2% | 61.5% | 46.2% | 1.000 |
| **Trauma** | 14 | 64.3% | 64.3% | 64.3% | 1.000 |

**Supplementary Table 2b.** Proportion of AI responses within 10 percentage points of the most popular clinician response, by orthopaedic subspecialty, with Cochran’s Q test p-values adjusted using a Bonferroni correction across the eight subspecialties.

| **Subspecialty** | **ChatGPT-5** | **Gemini 2.5 Flash** | **Grok-3** | **P value (Adjusted)** |
| --- | --- | --- | --- | --- |
| **Foot/Ankle** | 50.0% | 83.3% | 91.7% | 0.242 |
| **Hand** | 60.0% | 80.0% | 80.0% | 1.000 |
| **Knee/Sports** | 66.7% | 66.7% | 66.7% | 1.000 |
| **Paediatric** | 75.0% | 91.7% | 83.3% | 1.000 |
| **Reconstruction** | 63.6% | 72.7% | 72.7% | 1.000 |
| **Shoulder/Elbow** | 92.3% | 84.6% | 84.6% | 1.000 |
| **Spine** | 69.2% | 69.2% | 53.8% | 1.000 |
| **Trauma** | 64.3% | 64.3% | 64.3% | 1.000 |

**Supplementary Table 2c.** Proportion of AI responses within 20 percentage points of the most popular clinician response, by orthopaedic subspecialty, with Cochran’s Q test p-values adjusted using a Bonferroni correction across the eight subspecialties.

| **Subspecialty** | **ChatGPT-5** | **Gemini 2.5 Flash** | **Grok-3** | **P value (Adjusted)** |
| --- | --- | --- | --- | --- |
| **Foot/Ankle** | 66.7% | 91.7% | 100.0% | 0.594 |
| **Hand** | 80.0% | 90.0% | 90.0% | 1.000 |
| **Knee/Sports** | 100.0% | 100.0% | 91.7% | 1.000 |
| **Paediatric** | 100.0% | 100.0% | 83.3% | 1.000 |
| **Reconstruction** | 72.7% | 81.8% | 81.8% | 1.000 |
| **Shoulder/Elbow** | 92.3% | 92.3% | 92.3% | 1.000 |
| **Spine** | 69.2% | 69.2% | 61.5% | 1.000 |
| **Trauma** | 71.4% | 71.4% | 71.4% | 1.000 |
